# A Negative Feedback Model to Explain Regulation of SARS-CoV-2 Replication and Transcription

**DOI:** 10.3389/fgene.2021.641445

**Published:** 2021-02-26

**Authors:** Xin Li, Zhi Cheng, Fang Wang, Jia Chang, Qiang Zhao, Hao Zhou, Chang Liu, Jishou Ruan, Guangyou Duan, Shan Gao

**Affiliations:** ^1^College of Life Sciences, Nankai University, Tianjin, China; ^2^The Second Hospital of Tianjin Medical University, Tianjin, China; ^3^State Key Laboratory of Medicinal Chemical Biology, Nankai University, Tianjin, China; ^4^School of Mathematical Sciences, Nankai University, Tianjin, China; ^5^School of Life Sciences, Qilu Normal University, Jinan, China

**Keywords:** coronavirus, transcription, replication, regulation model, nsp15

## Abstract

**Background:**

Coronavirus disease 2019 (COVID-19) is caused by severe acute respiratory syndrome coronavirus 2 (SARS-CoV-2). Although a preliminary understanding of the replication and transcription of SARS-CoV-2 has recently emerged, their regulation remains unknown.

**Results:**

By comprehensive analysis of genome sequence and protein structure data, we propose a negative feedback model to explain the regulation of CoV replication and transcription, providing a molecular basis of the “leader-to-body fusion” model. The key step leading to the proposal of our model was that the transcription regulatory sequence (TRS) motifs were identified as the cleavage sites of nsp15, a nidoviral RNA uridylate-specific endoribonuclease (NendoU). According to this model, nsp15 regulates the synthesis of subgenomic RNAs (sgRNAs), and genomic RNAs (gRNAs) by cleaving TRSs. The expression level of nsp15 controls the relative proportions of sgRNAs and gRNAs, which in turn change the expression level of nsp15 to reach equilibrium between the CoV replication and transcription.

**Conclusion:**

The replication and transcription of CoVs are regulated by a negative feedback mechanism that influences the persistence of CoVs in hosts. Our findings enrich fundamental knowledge in the field of gene expression and its regulation, and provide new clues for future studies. One important clue is that nsp15 may be an important and ideal target for the development of drugs (e.g., uridine derivatives) against CoVs.

## Introduction

**FIGURE 1:**
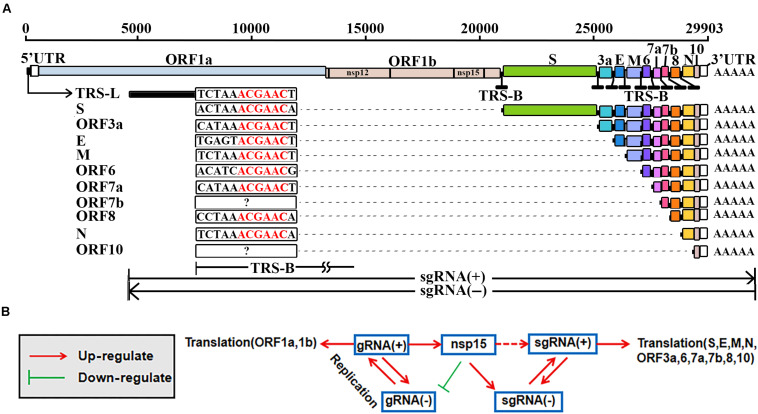
Replication and transcription of SARS-CoV-2. The elements used to represent the SARS-CoV-2 genome (GenBank: MN908947.3) were originally used in the previous study (Kim D. et al., 2020). TRS-L, transcription regulatory sequence in the leader; TRS-B, transcription regulatory sequence in the body; gRNA(+), genomic RNA; gRNA(–), antisense genomic RNA; sgRNA(+), subgenomic RNA; sgRNA(–), antisense subgenomic RNA; nsp12, RNA-dependent RNA polymerase (RdRP); nsp15, nidoviral RNA uridylate-specific endoribonuclease (NendoU). **(A)** The TRS-Bs of different genes have different lengths ranging from 6 nt to more than 100 nt. The *ORF7b* and *ORF10* genes may contain non-canonical TRS motifs (noted by?). **(B)** Nsp15 may also cleavage at ICSs on the sense strands, resulting in sgRNAs(+). Molecular experiments need be conducted to verify the possible cleavage at ICSs (noted by a dashed line).

Coronavirus disease 2019 (COVID-19) (Shah and Khan, 2020; Tahir et al., 2020) is caused by severe acute respiratory syndrome coronavirus 2 (SARS-CoV-2). As enveloped viruses which are composed of positive-sense and single-stranded RNAs, coronaviruses (CoVs) have the largest genomes (26–32 kb) among all RNA virus families. SARS-CoV-2 has a genome of ∼30 kb (Jiayuan et al., 2020), including 12 genes that are *ORF1a*, *1b*, *spike (S)*, *envelope (E)*, *membrane (M)*, *nucleocapsid (N)*, *ORF3a*, *6*, *7a*, *7b*, *8*, and *10*. The *ORF1a* and *1b* genes encode 16 non-structural proteins, named from nsp1 to nsp16. Among nsp1–16, nsp12, and nsp15 (Figure 1A) are RNA-dependent RNA polymerase (RdRP) (Hillen et al., 2020) and nidoviral RNA uridylate-specific endoribonuclease (NendoU) (Kim Y. et al., 2020), respectively. The other 10 genes encode four structural proteins (S, E, M, and N) and six accessory proteins (ORF3a, 6, 7a, 7b, 8, and 10) that are yet to be experimentally verified.

The genomic RNA, as a positive-sense RNA [gRNA(+)], is used as a template for the translation of nsp1–16 (da Silva et al., 2020), while the translation of 10 other proteins (S, E, M, N, ORF3a, 6, 7a, 7b, 8, and 10) of SARS-CoV-2 can be explained by the prevailing “leader-to-body fusion” model (Sawicki and Sawicki, 1998). In this model (Figure 1A), the replication and transcription of CoVs require gRNAs(+) as templates for the synthesis of antisense genomic RNAs [gRNAs(−)] and antisense subgenomic RNAs [sgRNAs(−)] by RdRP. When RdRP pauses, as it crosses a body transcription regulatory sequence (TRS-B) and switches the template to the leader TRS (TRS-L), sgRNAs(−) are formed through the discontinuous transcription (also referred to as polymerase jumping or template switching). Otherwise, RdRP reads gRNAs(+) continuously, without interruption, resulting in gRNAs(−). Thereafter, gRNAs(−) and sgRNAs(−) are used as templates to synthesize gRNAs(+) and sgRNAs(+), respectively; sgRNAs(+) are used as templates for the translation of the 10 proteins mentioned above. The “leader-to-body fusion” model may be at least partially conserved in coronaviridae (Kim D. et al., 2020). Although a three-step working model has been proposed to explain the underlying mechanisms in a previous study (Zuniga et al., 2004), the molecular basis of the “leader-to-body fusion” model remains unknown.

Recently, the “leader-to-body fusion” model in SARS-CoV-2 was directly validated in a study (Kim D. et al., 2020) using Nanopore RNA-seq – a direct RNA sequencing method (Xu et al., 2019). By reanalysis of the Nanopore RNA-seq data, we found a 6-nt TRS motif ACGAAC in the junction regions between TRS-Bs and the TRS-L of SARS-CoV-2 (Figure 1A). By comprehensive analysis of genome sequence and protein structure data, we identified GTTCGT as a motif of nsp15 cleavage sites, providing a clue to better understand the “leader-to-body fusion” model. In the present study, we aimed to determine the molecular basis of the “leader-to-body fusion” model and construct a model to explain the regulation of SARS-CoV-2 replication and transcription.

## Results

### TRS Motifs in Leader-Body Junction Regions

Using the Nanopore RNA-seq data in the previous study (Kim D. et al., 2020), 575,106 out of 879,679 reads from a SARS-CoV-2-infected Vero cell sample were aligned to the SARS-CoV-2 reference genome (GenBank: MN908947.3). Among all aligned reads, 575,106 sense reads represented gRNAs(+) or sgRNAs(+), while 30 antisense reads represented gRNAs(−) or sgRNAs(−). Using the next-generation sequencing (NGS) data in the previous study (Kim D. et al., 2020), 198,198,542 contiguous and 11,820,438 junction-spanning reads, representing gRNAs(+)/gRNAs(−) and sgRNAs(+)/sgRNAs(−), respectively, were aligned to the SARS-CoV-2 reference genome. The high ratio (198,198,542 vs. 11,820,438) between gRNAs(+)/gRNAs(−) and sgRNAs(+)/sgRNAs(−) was reported in that previous study (Kim D. et al., 2020), indicating that “leader-to-body fusion” does not have a high success rate. The exceedingly high ratio (575,106 vs. 30) between gRNAs(+)/sgRNAs(+) and gRNAs(−)/sgRNAs(−) may be mainly due to the differences of their degradation efficiencies. This ratio, however, was not noticed in that previous study (Kim D. et al., 2020).

A TRS-L usually comprises the first 60∼70 nts of the 5′ UTR in a CoV genome, while TRS-Bs with varied lengths are located immediately upstream of ORFs except ORF1a and 1b (Figure 1A). A motif in the junction regions between TRS-Bs and the TRS-L is defined as a TRS motif. By reanalysis of the Nanopore RNA-seq data, we found a 6-nt TRS motif ACGAAC in the junction regions between TRS-Bs and the TRS-L of SARS-CoV-2 (Figure 1A). Then, we defined the junction regions of eight genes (*S, E, M, N, ORF3a, 6, 7a*, and *8*) containing the canonical TRS motif ACGAAC (Table 1) as canonical junction regions. Accordingly, the sgRNAs(+) and sgRNAs(−) containing the canonical junction regions were defined as canonical sgRNAs(+) and sgRNAs(−), respectively. As the possible junction regions of *ORF7b* and *ORF10* exhibited high diversity in their sequences, they may contain multiple non-canonical TRS motifs ACGNNN or AAGNNN. Each of these non-canonical TRS motifs, however, was only supported by a few junction-spanning reads.

**TABLE 1:** TRS motifs in SARS-CoV-2 and SARS-CoV.

Motif	Type	Position	SARS2(Start-End)	Position	SARS1(Start-End)
GTTCGT	ICS	16014	nsp12(13483-16236)	3687	nsp3(3011-9962)
	ICS	28198	ORF8(27894-28259)	15928	nsp12(13397-16150)
	ICS	28233	ORF8(27894-28259)	18200	nsp14(17963-19739)
				18499	nsp14(17963-19739)
				22091	S(21476-25243)
				28415	N(28133-29401)
ACGAAC	TRS-L	70	5′ UTR(1-265)	51	5′ UTR(1-248)
	TRS-B	21556	S(21563-25384)	21470	S(21476-25243)
	TRS-B	25385	ORF3a(25393-26220)	25244	ORF3a(25252-26076)
	TRS-B	26237	E(26245-26472)	26093	E(26101-26331)
	TRS-B	26473	M(26523-27191)	26332	M(26382-27047)
	TRS-B	27041	ORF6(27202-27387)	26897	ORF6(27058-27249)
	TRS-B	27388	ORF7a(27394-27759)	27251	ORF7a(27257-27625)
	TRS-B	27888	ORF8(27894-28259)	27757	ORF8(27763-28131)
	TRS-B	28260	N(28274-29533)	28119	N(28133-29401)
*			ORF7b		ORF7b
*			ORF10		ORF10

ACGAAC and GTTCGT (in the first column) are used to represent the motifs of nsp15 cleavage sites on the antisense and sense strands, respectively. The motifs were aligned to the SARS-CoV-2 genome (GenBank: MN908947) and the SARS-CoV genome (GenBank: AY278489) using positions (in the third and fifth columns) of their first nucleotides. The fourth and sixth columns show the regions influenced by the motifs in the third and fifth columns, respectively. Nsp3 is encoded by the *ORF1a* gene; nsp12 (RdRP) and nsp14 are encoded by the *ORF1b* gene (see section “Introduction”). * the possible junction regions of *ORF7b* and *ORF10* may contain non-canonical TRS motifs. ICS, intragenic cleavage site; TRS-L, transcription regulatory sequence in the leader; TRS-B, transcription regulatory sequence in the body.

### Molecular Basis of “Leader-to-Body Fusion” Model

Further analysis of 1,265 betacoronaviruses (see section “Materials and Methods”) showed that ACGAAC is highly conserved in the predicted TRS-Bs of CoV genomes from subgroup B, C, and D (Duan et al., 2020), while the TRS motif is ACTTTA in CoV genomes from subgroup A. This suggests that TRS motifs or their reverse complimentary sequences are necessary for enzyme recognition or processing. Since the “leader-to-body fusion” model may be at least partially conserved in coronaviridae (Kim D. et al., 2020), the corresponding enzymes should be encoded by the *ORF1a* or *1b* gene, given their likelihood to be translated. After analyzing nsp1–16 encoded by the *ORF1a* and *1b* genes (see section “Introduction”), we determined that nsp15 is most likely to function in these junction regions, given that a homolog of nsp15 has cleavage sites containing at least one uridine (Nedialkova et al., 2009). By comprehensive analysis of genome sequence and protein structure data, we determined that the cleavage sites of nsp15 is likely to contain the motif “GTTCGT| N” [the vertical line indicates the breakpoint and N indicates any nucleotide base (Nedialkova et al., 2009)], read on the antisense strands of CoV genomes. Thus, ACGAAC and GTTCGT can be used to represent the motifs of nsp15 cleavage sites on the antisense and sense strands, respectively.

Upon searching for ACGAAC and GTTCGT in the genomes of betacoronavirus subgroup B, the occurrence of ACGAAC was found to be more than 1.6 times that of GTTCGT. In particular, ACGAAC and GTTCGT occurred nine and three times (Table 1) in the SARS-CoV-2 genome, respectively. These findings suggest that the differences of the degradation efficiencies between sense and antisense reads (see above) result from substantially more cleavage of gRNAs(−)/sgRNAs(−) than that of gRNAs(+)/sgRNAs(+). Here, we do not rule out other explanations for this high ratio. For example, gRNAs(+)/sgRNAs(+) are protected by binding to the N proteins. Based on the above analyses, we proposed a molecular basis of the “leader-to-body fusion” model. In our proposal, the basic function of nsp15 involves in the degradation of gRNAs and sgRNAs. Nsp15 cleaves gRNAs(−) and sgRNAs(−) at TRS-Bs(−). Then, the free 3′ ends (∼6 nts) of TRS-Bs(−) hybridize the junction regions of TRS-Ls for template switching (see above). Nsp15 also cleaves gRNAs(−) and sgRNAs(−) at TRS-Ls(−), which is not necessary for template switching. The probability of successful hybridization is prone to many factors, which explains the presence of sgRNAs without TRS-Ls reported in the previous study (Kim D. et al., 2020). The sgRNAs(−) without TRS-Ls degrade quickly, which explains the high ratio (see above) between gRNAs(+)/gRNAs(−) and sgRNAs(+)/sgRNAs(−). In addition, some non-canonical sgRNAs(+) and sgRNAs(−) are synthesized as a result of occasional hybridization between the free 3′ ends of TRS-Bs(−) and highly similar sequences of TRS-L junction regions, which explains the presence of non-canonical sgRNAs(+) and sgRNAs(−) reported in the previous study (Kim D. et al., 2020).

**FIGURE 2:**
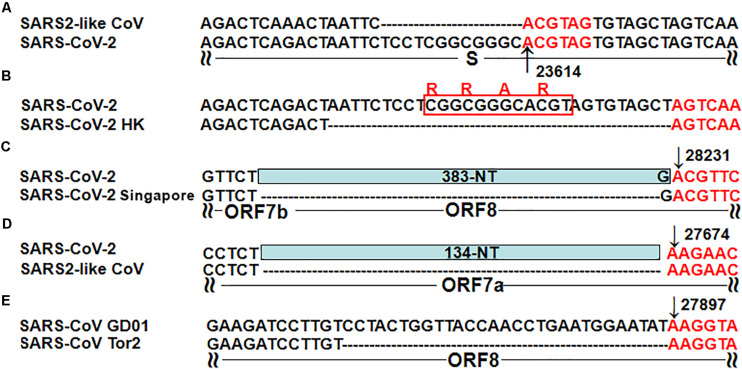
Five typical recombinant events in SARS-CoV-2 and SARS-CoV. **(A)** The genome sequences are from the SARS-like CoV strain WIV1 from bats (GenBank: KF367457) and SARS-CoV-2 (GenBank: MN908947); **(B)** The genome sequences are from SARS-CoV-2 (GenBank: MN908947) and the SARS-CoV-2 strain Hong Kong (GISAID: EPI_ISL_417443); **(C)** The genome sequences are from SARS-CoV-2 (GenBank: MN908947) and the SARS-CoV-2 strain Singapore (GISAID: EPI_ISL_414378, EPI_ISL_414379 and EPI_ISL_414380); **(D)** The genome sequences are from SARS-CoV-2 (GenBank: MN908947) and the mink SARS2-like CoV strain (GenBank: MT457390); **(E)** The genome sequences are from the SARS-CoV strain GD01 (GenBank: AY278489) and the SARS-CoV strain Tor2 (GenBank: AY274119).

Nsp15 cleavage may also occur at intragenic sites containing GTTCGT (defined as intragenic cleavage sites – ICSs), which, however, has not been reported, as far as we know. Therefore, molecular experiments need be conducted to verify the nsp15 cleavage at ICSs on the sense strands of CoVs. Among the three ICSs in the SARS-CoV-2 genome (Table 1), one is located in the coding sequence (CDS) of RdRP, while the other two are located in the *ORF8* gene. These two ICSs are also present in the *ORF8* genes of most SARS2-like CoV and SARS-like CoV genomes; however, they are absent in the SARS-CoV genomes from humans (GenBank: AY274119 and AY278489) and the SARS-like CoV genomes from civets (GenBank: AY304486, AY515512, and AY572034). One of the two ICSs is present in the genome of the SARS-like CoV strain WIV1 from bats (GenBank: KF367457), which was considered most closely related to SARS-CoV (Xing-Yi et al., 2013). Deletions of *ORF8* (Figure 2) were reported to be associated with attenuation of SARS-CoV (Muth et al., 2018) and SARS-CoV-2 (Su et al., 2020). The *ORF8* gene of SARS-CoV is considered to have played a significant role in adaptation to human hosts following interspecies transmission via the modification of viral replication (Lau et al., 2015). The loss of two nsp15 ICSs in *ORF8* may account for the enhanced functions of *ORF8* in the SARS-CoV genome.

The molecular basis of “leader-to-body fusion” model can also be used to explain recombination events in betacoronavirus genomes, particularly five typical recombination events reported in the previous studies. By analysis of both canonical and non-canonical TRS motifs, we determined that the first three nucleotides of these recombination sites are in favor of AAG or ACG. The first recombination site (Figure 2A) was a 12-nt insertion in the SARS-CoV-2 genome, which formed a furin cleavage site (FCS) “RRAR” in the junction region between S1 and S2 subunits of the S protein (Li et al., 2020). This novel junction FCS is only present in SARS-CoV-2 among all betacoronaviruses of subgroup B, which suggests that it was acquired through “leader-to-body fusion.” The second recombination site (Figure 2B) was a 30-nt deletion, resulting in loss of the novel junction FCS in the SARS-CoV-2 strain Hong Kong. This attenuated strain appeared soon during the early stages of human-to-human transmission as we predicted (Li et al., 2020). The third recombination site (Figure 2C) was a 383-nt deletion spanning the *ORF7b* and *ORF8* genes of the SARS-CoV-2 strain Singapore (Su et al., 2020). The fourth recombination site (Figure 2D) was a 134-nt deletion in the *ORF7a* gene of mink SARS2-like CoV that is a variant strain of SARS-CoV-2. The fifth recombination site (Figure 2E) was a 29-nt deletion in the *ORF8* gene of the SARS-CoV strain Tor2 (Muth et al., 2018). Furthermore, we found two ACGAAC motifs closely flanking *ORF8* upstream and downstream (Table 1), which suggests that *ORF8* was acquired by betacoronavirus subgroup B through “leader-to-body fusion.” As the most important genomic features of SARS-CoV-2 and SARS-CoV, the novel junction FCS and the enhanced *ORF8* were identified to provide clues for the future study of their origin and evolution, respectively.

### Proposal of a Negative Feedback Model

Based on the above analyses, we propose a negative feedback model (Figure 1B) to explain the regulation of CoV replication and transcription. In this model, nsp15 regulates the synthesis of sgRNAs or gRNAs by the cleavage of TRS-Bs(−). The expression level of nsp15 controls the relative proportions of sgRNAs and gRNAs. An increase of nsp15 expression results in fewer gRNAs(−) and more sgRNAs(−), after which fewer gRNAs(+) and more sgRNAs(+) are synthesized, respectively. A decrease of gRNAs(+) results in a decrease of nsp15 expression, given that nsp15 is translated using gRNA(+) as the template. Furthermore, the nsp15 ICS in the CDS of RdRP (Table 1) may enhance the negative feedback regulation. Via negative feedback regulation, CoVs reach equilibrium between the replication and transcription (Figure 1B); thus, this mechanism may be important for the persistence of CoVs in hosts.

Our negative feedback model is based on the determination of the molecular basis of the “leader-to-body fusion” model. Our hypothesis is that the “leader-to-body fusion” of CoVs dose not rely on the genes of hosts, which is different from the models proposed in the previous studies (Zuniga et al., 2004). The key step leading to the proposal of our model was that the TRS motifs were identified as the cleavage sites of nsp15, mainly due to the integration of information from many aspects, particularly considering: (1) the ratio between sense and antisense reads, and the ratio between contiguous and junction-spanning reads (see above); (2) the identification of canonical and non-canonical TRS motifs; (3) the nsp15 structure in complex with GpU (PDB: 6X1B); (4) the nsp15 ICSs in *ORF8* (see above); (5) the polyT at the tail of “GTTCGT” or polyA before “ACGAAC,” which ensures the presence of at least one uridine for nsp15 cleavage.

### The Necessity of Negative Feedback Regulation

**FIGURE 3:**
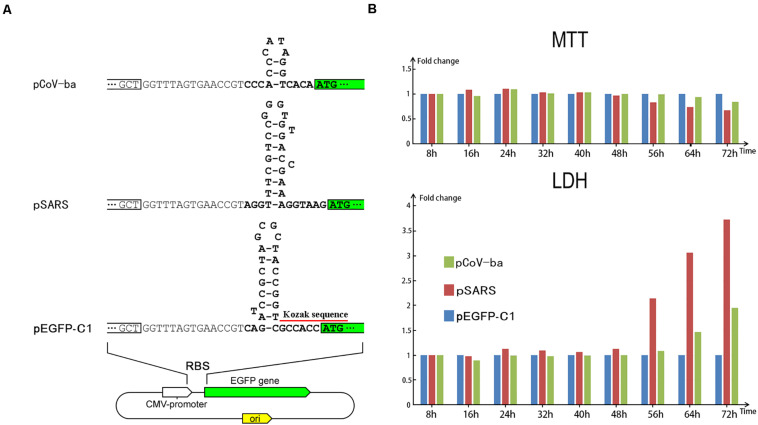
Regulation of EGFP translation by three types of plasmids. **(A)** The only differences among the three types of plasmids (pEGFP-C1, pSARS, and pCoV-ba) are their 29-, 31-, and 17-nt inserts (Supplementary 1) forming different hairpins (see section “Materials and Methods”). **(B)** HEK293 cells were transfected with three types of plasmids. At each time after transfection, the viability (MTT) and the LDH release were measured using MTT and LDH, respectively. Used as control, the measured values of cells transfected by pEGFP-C1 were set to 1. Then, the relative viability (MTT) and the relative LDH release of cells transfected by pSARS and pCoV-ba are represented on the *y*-axis.

To indirectly prove that the negative feedback regulation is important for the persistence of CoVs in hosts, we designed preliminary experiments to examine the cellular effect caused by over-expression of exogenous genes without negative feedback regulation. In our previous study (Duan et al., 2020), we proposed that the first hairpin (immediately upstream of the first gene *ORF1a*) has an important role in the functions (e.g., regulation of translational initiation) of the ribosome binding site (RBS) in the 5′ UTR of the SARS-CoV-2 genome. First hairpins with proper structures (Figure 3A) may enhance the translation of their downstream genes. This inspired us to use the first hairpins of CoVs in designing experiments for the over-expression of *EGFP*. Based on our hypothesis (Duan et al., 2020), betacoronavirus subgroup B (including SARS-CoV and SARS-CoV-2) shares an first hairpin, which is theoretically able to up-regulate the translation of downstream genes, while betacoronavirus subgroup A shares another first hairpin, which is unlikely to up-regulate the translation of downstream genes. This difference between first hairpins of betacoronavirus subgroup A and B was used in designing plasmids for comparative tests.

In total, we designed three types of plasmids (Figure 3A) containing *EGFP* as reporter genes – named pEGFP-C1, pSARS, and pCoV-ba (ba represents betacoronavirus subgroup A). The plasmid pEGFP-C1 used as control contains a 29-nt sequence, forming the first hairpin of cytomegalovirus (CMV). Two other types of plasmids proceeded by 31- and 17-nt inserts (Supplementary 1) were constructed to change the expression of *EGFP* (see section “Materials and Methods”). The 31- and 17-nt inserts form the first hairpins of betacoronavirus subgroup B and A, respectively. Comparing the fluorescent brightness of cells transfected with three types of plasmids, the translation of *EGFP* in pSARS was markedly higher than that in pEGFP-C1 and pCoV-ba (Supplementary 1). Moreover, significant cell death was observed in samples transfected by pSARS using a microscope. We then performed 3-(4,5-dimethyl-2-thiazolyl)-2,5-diphenyl-2-H-tetrazolium bromide (MTT) and lactate dehydrogenase (LDH) experiments to further examine the cellular effect of plasmid transfection (see section “Materials and Methods”). Both MTT and LDH experiments consistently demonstrated that pSARS caused significantly more HEK293T and HeLa cell death at 48 h after its transfection, than pEGFP-C1 and pCoV-ba. To determine whether three types of plasmids change the expression of *EGFP* mainly at the translation level and rule out other possible factors that may exert influence at the replication or transcription level, we performed additional experiments: (1) using HEK293 cells to rule out the possible influence by the differences of plasmid copy numbers, since all three types of plasmids containing the SV40 origins can be replicated to copy numbers of between 400∼1000 plasmids per cell in HEK293T cells; and (2) using qPCR to rule out the possible influence by differential transcription of *EGFP*. The results of additional experiments showed: (1) at 56 h after transfection of pSARS, the relative viability (MTT) decreased to 83.46% and the relative LDH release increased to 2.14 fold (Figure 3B) in HEK293 cells; and (2) significant differential transcription of *EGFP* was not detected by qPCR in HEK293 cells (Supplementary 1). Given that the only differences among the three types of plasmids are their 29-, 31-, and 17-nt inserts forming different hairpins, we concluded that these hairpins change the expression of *EGFP* mainly at the translation level. The hairpin in pSARS resulted in the over-expression of *EGFP*, which caused more cell death. These results showed that over-expression of exogenous genes without negative feedback regulation causes cell death.

## Conclusion and Discussion

In the present study, we propose a negative feedback model to explain the regulation of SARS-CoV-2 replication and transcription, providing a molecular basis of the “leader-to-body fusion” model. The key step leading to the proposal of our model was that the TRS motifs were identified as the cleavage sites of nsp15. Our model does not rule out other RNAs or proteins involved in the relevant functions. The template switching ability and the high ratio between contiguous and junction-spanning reads suggested that RdRP has high enzyme activity. Relatively few junction-spanning reads indicated that the nsp15-induced “leader-to-body fusion” does not have a high success rate. This suggests that nsp15 is an important and ideal target for the development of drugs against CoVs. The most recent nsp15 structure in complex with GpU (PDB: 6X1B) shows that the uridine binds to the active site of nsp15. Thus, uridine derivatives, such as Tipiracil, Uridine-5′-Monophosphate, Uridine-3′-Monophosphate, citrate, Trifluridine, Tegafur, Carmofur, Furtulon, etc., are potential inhibitors of this enzyme.

The hairpins immediately upstream of the first genes (first hairpins) play an important role in the regulation of CoV gene expression. The results of preliminary experiments showed that the first hairpin in pSARS resulted in the over-expression of *EGFP*, which caused cell death. Without negative feedback regulation, over-expression of exogenous genes causes cell death. The negative feedback regulation may be important for the persistence of CoVs in hosts. However, whether first hairpins change the expression of *EGFP* mainly at the translation level is still undetermined, since we only used plasmid DNA, rather than mRNA in the experiments. As an additional finding, the Kozak consensus sequence GCCACCAUGG (Figure 1B) is not necessary for protein translation in eukaryotes. These findings can be used to design vaccine, drug and expression vectors.

Our findings enrich fundamental knowledge in the field of gene expression and its regulation, and provide new clues for future studies. However, current studies involving NendoU remain contradictory in terms of their findings, regarding fundamental questions: (1) NendoU is conserved among coronaviruses, arteriviruses and toroviruses, is it present in non-vertebrate-infecting representatives of the nidoviruses order? (2) is nsp15 indispensable for viral replication and living? (3) is nsp15 responsible for protein interference with the innate immune response? and (4) Under what conditions does nsp15 cleave the targets, particularly from hosts? The discovery of more nsp15 cleavage sites in viral or host genomes will provide new clues to answer these questions.

## Materials and Methods

1,265 genome sequences of betacoronaviruses (in subgroups A, B, C, and D) were downloaded from the NCBI Virus database^1^ in our previous study (Duan et al., 2020). Among these genomes, 292 belongs to betacoronavirus subgroup B (including SARS-CoV and SARS-CoV-2). Nanopore RNA-seq data was downloaded from the website^2^ for the reanalysis of leader-body junction regions. The results were confirmed using Illumina RNA-seq data from the NCBI SRA database under the accession number SRP251618. Data cleaning and quality control were performed using Fastq_clean (Zhang et al., 2014). Statistics and plotting were conducted using the software R v2.15.3 with the Bioconductor packages (Gao et al., 2014). The 5′ and 3′ ends of gRNAs(+) and sgRNAs(+) were observed and double-checked using the software Tablet v1.15.09.01 (Milne et al., 2013).

In the present study, three types of plasmids (pEGFP-C1, pSARS, and pCoV-ba), and three types (HeLa, HEK293T, and HEK293) of cells were used for transfection. pEGFP-C1 was maintained in our lab. To construct pSARS, pEGFP-C1 was PCR amplified using primers fVR and rRBS2 (Supplementary 1). Then, the linear PCR product was self-ligated into a plasmid by homologous recombination technology using ClonExpress II One Step Cloning Kit (Vazyme Biotech, China). Following the same procedure, primers fVR and rRBS3 (Supplementary 1) were used to construct pCoV-ba. HeLa, HEK293T, and HEK293 were cultured in Dulbecco’s Modified Eagle Medium (DMEM) media supplemented with 10% fetal bovine serum. About 100,000 cells were seeded into one well of a 6-well plate for plasmid transfection. After 12 h (this time was set as 0 h in Figure 2B), the medium was changed and 1 μg of plasmid DNA was transfected into one well using 3 μL PolyJet (SignaGen Laboratories, United States), according to the manufacturer’s instruction. At 8 h after the 0 h, the medium was changed until the MTT or LDH measurement. MTT (5 mg/mL × 20 μL) and 180 μL medium were added to 5,000 cells and cultured at 37°C for 4 h for each measurement. Next, the cells were removed of medium, washed with PBS, then mixed with 100 μL DMSO to dissolve the formazan product. Finally, formazan absorbance was measured by a microplate reader with a wavelength of 492 nm (Thermo Labsystems, Helsinki, Finland). LDH experiments were performed using LDH cytotoxicity assay detection kit (Beyotime, China), according to the manufacturer’s instruction.

## Data Availability Statement

The original contributions presented in the study are included in the article/Supplementary Material, further inquiries can be directed to the corresponding authors.

## Author Contributions

SG conceived the project and drafted the main manuscript text. SG and GD supervised this study. GD and JC performed programming. ZC, HZ, and FW conducted the experiments. XL and QZ downloaded, managed, and processed the data. SG, CL, and JR revised the manuscript. All authors contributed to the article and approved the submitted version.

## Conflict of Interest

The authors declare that the research was conducted in the absence of any commercial or financial relationships that could be construed as a potential conflict of interest.

## Footnotes

1 https://www.ncbi.nlm.nih.gov/labs/virus

2 https://osf.io/8f6n9/files/

## Supplementary Material

Supplementary Figure 1 pEGFP-C1 plasmid information.

Supplementary Figure 2 Comparison of fluorescent brightness.

Supplementary Table 1 Primers for plasmid construction.
