# Supplementary material for: A Negative Feedback Model to Explain Regulation of SARS-CoV-2 Replication and Transcription
Source: Front Genet. 2021 Feb 26;12:641445. doi: 10.3389/fgene.2021.641445 (PMC7954359; doi:10.3389/fgene.2021.641445)
Supplement: Supplementary Figure 1 — pEGFP-C1 plasmid information. [file Table_1.DOC]

# Supplementary 1

### **1.1 Construction of plasmids**

Table S1. Primers for plasmid construction

| **Name** | **Forward primer (5'-3')**  **Reverse primer (5'-3')** |
| --- | --- |
| pSARS | ACGGTTCACTAAACCAGCTC (fVR) |
|  | GAGCTGGTTTAGTGAACCGTAGGTTTCGTCCGGGTGTGAC  CGAAAGGTAAGATGGTGAGCAAGGGCGA (rRBS2) |
| pCoV-ba | ACGGTTCACTAAACCAGCTC (fVR) |
|  | GAGCTGGTTTAGTGAACCGTCCCACCCATAGGTCACAATG  GTGAGCAAGGGCGA (rRBS3) |

Based on pEGFP-C1, primers fVR and rRBS2 were used to construct pSARS, primers fVR and rRBS3 were used to construct pCoV-ba. The only differences among the three types of plasmids (pEGFP-C1, pSARS and pCoV-ba) are their 29-, 31- and 17-nt inserts: CAGATCCGCTAGCGCTACCGGTCGCCACC, AGGTTTCGTCCGGGTGTGACCGAAAGGTAAG and CCCACCCATAGGTCACA.


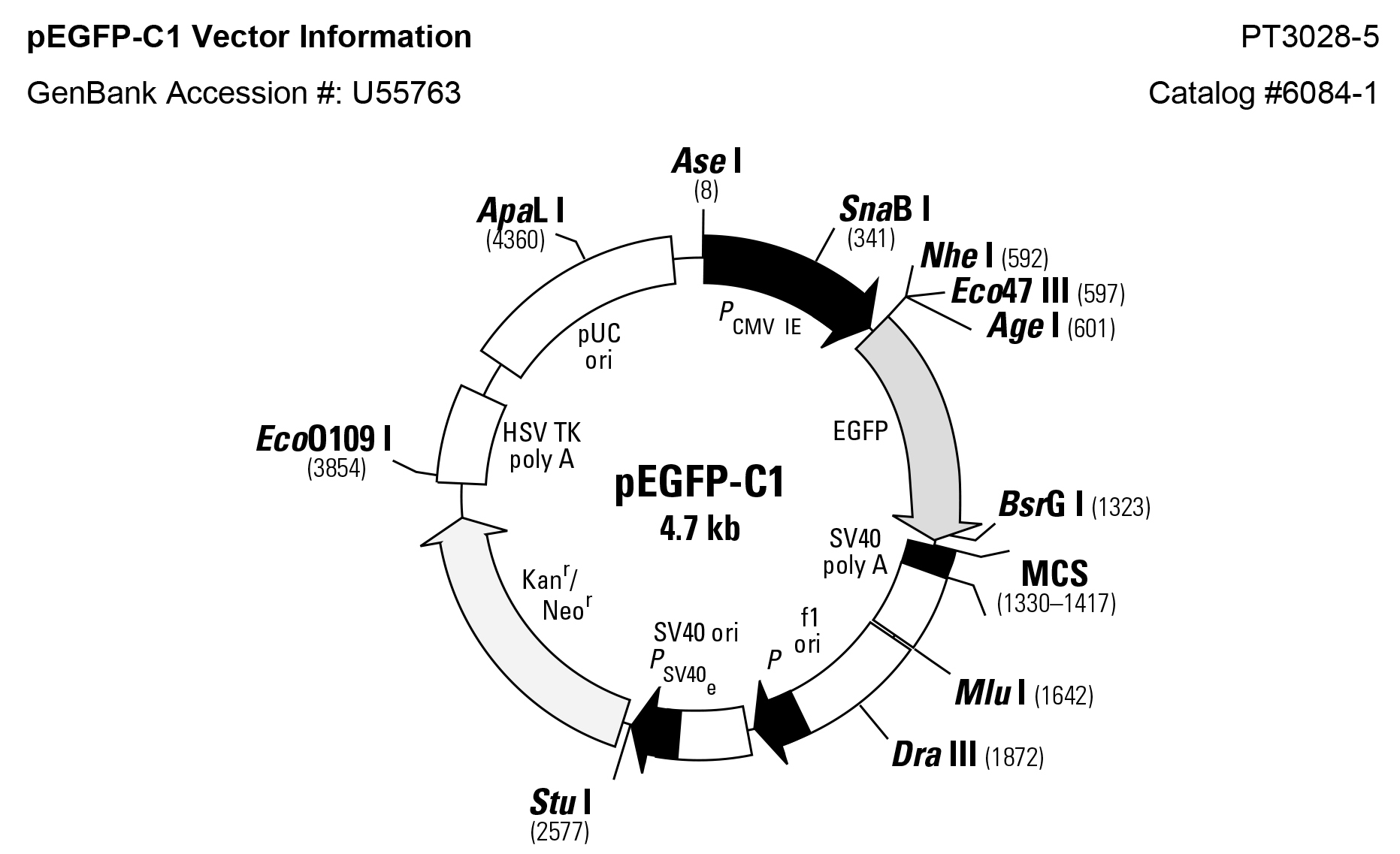


**Figure S2. pEGFP-C1 vector information.** This figure was originally used in the product description of Clontech Laboratories, Inc.

### **1.2 Comparison of fluorescent brightness**

| 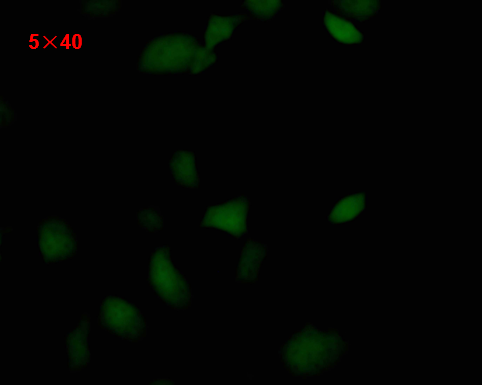 | 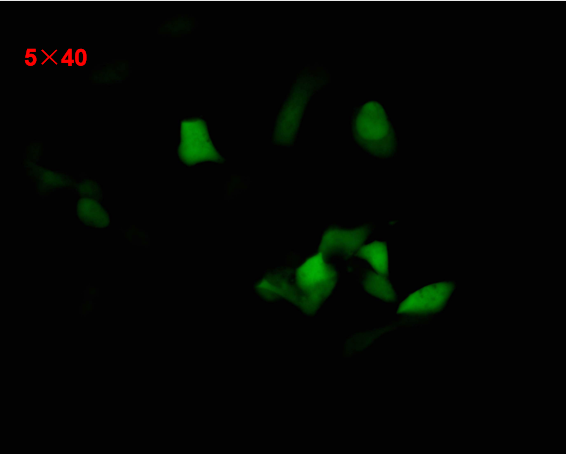 | 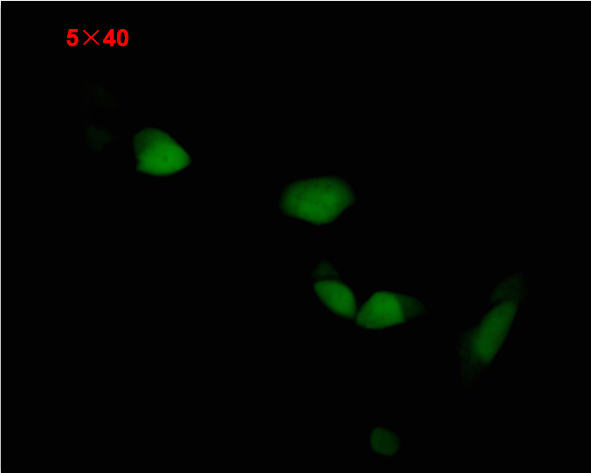 |
| --- | --- | --- |

**Figure S2. Comparison of fluorescent brightness.** HEK293 cells were observed by a fluorescent microscope WYS-41XDY(Tianjin Weiyi, China) with the same setting (exposure time: 200 ms, wavelength of the excitation laser: 488 nm) at the 48 hours after the transfection of three types of plasmids (from left to right pEGFP-C1, pSARS and pCoV-ba). By setting gray level of the black areas to 0, the average gray levels of the green areas for three subfigures are 41, 63 and 44.

### **1.3 Kits and procedures**

RNA extraction was performed using TRIzol Reagent (Thermo Scientific, USA) following the procedure provided by the company.

Using specific primers for the genes (e.g. EGFP and NeoR), PCR amplification coupled with Sanger sequencing was used to confirmed the targets.

The cDNA was performed using Thermo Scientific Revert Aid First Strand cDNA Synthesis Kit K1621 (Thermo Scientific, USA). PCR amplification was performed using LA Taq (TaKaRa, Japan). Each sample mixed with PCR reagent (**Table S3**) was incubated at 94 °C for 30 s, followed by 35 PCR cycles (10 s at 98 °C, 68 m at 68 °C, and 30 s at 72 °C for each cycle).

Table S3. PCR reagent for each sample

| TaKaRa LA Taq（5 U/μL） | 12.5 µL |
| --- | --- |
| dNTP Mixture（2.5 mM each） | 1.0 µL |
| Template | 1 µg |
| primer F (10pM/µL) | 1 µL |
| primer R (10pM/µL) | 1 µL |
| RNase/DNase Free Water | - |
| Total | 50 µL |

### **1.3 Primers for qPCR**

>EGFP

ATGGTGAGCAAGGGCGAGGAGCTGTTCACCGGGGTGGTGCCCATCCTGGTCGAGCTGGACGGCGACGTAAACGGCCACAAGTTCAGCGTGTCCGGCGAGGGCGAGGGCGATGCCACCTACGGCAAGCTGACCCTGAAGTTCATCTGCACCACCGGCAAGCTGCCCGTGCCCTGGCCCACCCTCGTGACCACCCTGACCTACGGCGTGCAGTGCTTCAGCCGCTACCCCGACCACATGAAGCAGCACGACTTCTTCAAGTCCGCCATGCCCGAAGGCTACGTCCAGGAGCGCACCATCTTCTTCAAGGACGACGGCAACTACAAGACCCGCGCCGAGGTGAAGTTCGAGGGCGACACCCTGGTGAACCGCATCGAGCTGAAGGGCATCGACTTCAAGGAGGACGGCAACATCCTGGGGCACAAGCTGGAGTACAACTACAACAGCCACAACGTCTATATCATGGCCGACAAGCAGAAGAACGGCATCAAGGTGAACTTCAAGATCCGCCACAACATCGAGGACGGCAGCGTGCAGCTCGCCGACCACTACCAGCAGAACACCCCCATCGGCGACGGCCCCGTGCTGCTGCCCGACAACCACTACCTGAGCACCCAGTCCGCCCTGAGCAAAGACCCCAACGAGAAGCGCGATCACATGGTCCTGCTGGAGTTCGTGACCGCCGCCGGGATCACTCTCGGCATGGACGAGCTGTACAAGTAA

F-CGACGTAAACGGCCACAAGT

R-TGCTGCTTCATGTGGTCGGG

>NeoR

ATGATTGAACAAGATGGATTGCACGCAGGTTCTCCGGCCGCTTGGGTGGAGAGGCTATTCGGCTATGACTGGGCACAACAGACAATCGGCTGCTCTGATGCCGCCGTGTTCCGGCTGTCAGCGCAGGGGCGCCCGGTTCTTTTTGTCAAGACCGACCTGTCCGGTGCCCTGAATGAACTGCAAGACGAGGCAGCGCGGCTATCGTGGCTGGCCACGACGGGCGTTCCTTGCGCAGCTGTGCTCGACGTTGTCACTGAAGCGGGAAGGGACTGGCTGCTATTGGGCGAAGTGCCGGGGCAGGATCTCCTGTCATCTCACCTTGCTCCTGCCGAGAAAGTATCCATCATGGCTGATGCAATGCGGCGGCTGCATACGCTTGATCCGGCTACCTGCCCATTCGACCACCAAGCGAAACATCGCATCGAGCGAGCACGTACTCGGATGGAAGCCGGTCTTGTCGATCAGGATGATCTGGACGAAGAGCATCAGGGGCTCGCGCCAGCCGAACTGTTCGCCAGGCTCAAGGCGAGCATGCCCGACGGCGAGGATCTCGTCGTGACCCATGGCGATGCCTGCTTGCCGAATATCATGGTGGAAAATGGCCGCTTTTCTGGATTCATCGACTGTGGCCGGCTGGGTGTGGCGGACCGCTATCAGGACATAGCGTTGGCTACCCGTGATATTGCTGAAGAGCTTGGCGGCGAATGGGCTGACCGCTTCCTCGTGCTTTACGGTATCGCCGCTCCCGATTCGCAGCGCATCGCCTTCTATCGCCTTCTTGACGAGTTCTTCTGA

F- TGCCTGCTTGCCGAATATCA

R- ATATCACGGGTAGCCAACGC
